# Bonded by nature: Humans form equally strong and reciprocated bonds with similar raised dogs and wolves

**DOI:** 10.3389/fpsyg.2022.1044940

**Published:** 2023-01-04

**Authors:** Megane E. Burkhard, Friederike Range, Samantha J. Ward, Lauren M. Robinson

**Affiliations:** ^1^Domestication Lab, Konrad Lorenz Institute of Ethology, University of Veterinary Medicine Vienna, Vienna, Austria; ^2^School of Animal Rural and Environmental Sciences, Nottingham Trent University, Nottingham, United Kingdom; ^3^Department of Psychology, University of Michigan, Ann Arbor, MI, United States; ^4^Language Research Center, Georgia State University, Atlanta, GA, United States

**Keywords:** canid, relationships, domestication, questionnaires, greeting

## Abstract

**Introduction:**

To explore human-canid relationships, we tested similarly socialized and raised dogs (*Canis familiaris*) and wolves (*Canis lupus*) and their trainers in a wildlife park. The aims of our study were twofold: first, we aimed to test which factors influenced the relationships that the trainers formed with the dogs or wolves and second, we investigated if the animals reacted to the trainers in accordance with the trainers’ perceptions of their relationship.

**Methods:**

To achieve these goals, we assessed the relationships using a human-animal bonds survey, which the trainers used to rate the bonds between themselves and their peers with the canids, and by observing dyadic trainer-canid social interactions.

**Results:**

Our preliminary results given the small sample size and the set-up of the research center, demonstrate that our survey was a valid way to measure these bonds since trainers seem to perceive and agree on the strength of their bonds with the animals and that of their fellow trainers. Moreover, the strength of the bond as perceived by the trainers was mainly predicted by whether or not the trainer was a hand-raiser of the specific animal, but not by whether or not the animal was a wolf or a dog. In the interaction test, we found that male animals and animals the trainers felt more bonded to, spent more time in proximity of and in contact with the trainers; there was no difference based on species.

**Discussion:**

These results support the hypothesis that wolves, similarly to dogs, can form close relationships with familiar humans when highly socialized (Canine Cooperation Hypothesis). Moreover, as in other studies, dogs showed more submissive behaviors than wolves and did so more with experienced than less experienced trainers. Our study suggests that humans and canines form differentiated bonds with each other that, if close, are independent of whether the animal is a wolf or dog.

## Introduction

The social relationship that exists between humans and other animals is believed to be thousands of years old (AVMA, 1998; Braje, 2011) and has been shown to have substantial effects on the welfare of both (Hosey et al., 2018). The oldest and probably the strongest human-animal relationship is that between humans and dogs (Topál et al., 1997; Payne et al., 2015). It is at the heart of the human-animal bond phenomenon (Nagasawa et al., 2009b; Fine and Beck, 2015) with bond being defined as ‘a mutually beneficial and dynamic relationship between people and animals that is influenced by behaviors essential to the health and wellbeing of both’ [(AVMA, 1998); see also (Hosey and Melfi, 2014)]. Multiple studies have demonstrated that humans can develop positive feelings and behaviors towards their dogs, creating a bond that has been compared to the one formed in human-infant relationships (Nagasawa et al., 2009b; Schneider et al., 2010; Udell and Brubaker, 2016). For example, at the behavioral level, humans tend to address and handle dogs and children in a similar way (Mitchell, 2001; Prato-Previde et al., 2003) and it has been shown that the limbic network (including the amygdala), which is thought to be involved in the activation of human attachment-related functions, is active when human mothers view images of their child and their dog (Stoeckel et al., 2014).

Dogs seem to reciprocate the positive relationships humans form with them by exhibiting affiliative behaviors which include, for instance, proximity and gaze seeking in stressful contexts (Cimarelli et al., 2016). These behaviors are particularly displayed towards the dog’s owner. For example, several studies found that, compared to strangers, dogs were more distressed when separated from their owner, and greeted and spent more time in contact with them [i.e., displaying more behaviors such as approaching, tail wagging, jumping and physical contact; Topál et al., 1998; Prato-Previde et al., 2003; Palmer and Custance, 2008; see (Payne et al., 2015) for a review]. This suggests that individual human-dog bonds differ depending on the dyad (Cimarelli et al., 2016). While it is still unclear whether the bond dogs form with their owner indeed constitutes attachment, e.g., relies on the same neural networks associated with attachment-related processes in humans, it is clear that dogs form a close affectionate bond with their owners (Karl et al., 2020). The fact that there are certain behaviors displayed specifically to certain humans (owner/caretaker) suggest that these behaviors are not just the consequence of socialization *per se* – e.g. the process of forming habits when interacting with the environment through education and training.

Thus, while it is clear that humans and dogs can form intricate affectionate bonds with each other, it is still a question whether this is specific to dogs, or rather based on the experience the animals and the caretakers have with each other. One approach to investigate this question is to compare the relationship humans form with dogs and those they form with wolves when having the same extensive experiences with the animals.

Studying wolves and dogs constitutes a very interesting comparison since, while closely related (Lindblad-Toh et al., 2005; Thalmann et al., 2013) and still quite similar in behavior [see (Range and Marshall-Pescini, 2022)], during domestication, dogs have been selected to live in the human environment (Miklósi and Topál, 2013; Range and Marshall-Pescini, 2022). This selection may have enabled them to expand their species-specific social–emotional behaviors [Emotional reactivity hypothesis (Hare and Tomasello, 2005a,b)] to form attachment bonds with humans [attachment hypothesis (Topál et al., 2005)] or be overall more social [Hypersociability hypothesis (Bentosela et al., 2016; von Holdt et al., 2017)]. While studies found that extensively human-socialized wolves are capable of forming long-lasting attachment bonds with their caregivers (Hall et al., 2015; Ujfalussy et al., 2017; Lenkei et al., 2020; Wheat et al., 2020), dogs do seem to be overall more social towards humans than wolves (Bentosela et al., 2016; von Holdt et al., 2017; Lazzaroni et al., 2020; Wirobski et al., 2021). Furthermore, being aware of these differences and the fact that wolves are highly successful group-hunting predators potentially dangerous to man (Linnell et al., 2002), might influence the attitude and thus relationships people engage in with wolves.

In a first step to investigate this topic, we set out to study the bond human caretakers form with dogs and wolves housed at a wildlife park. At the Wolf Science Center (WSC), wolves and dogs are handraised by human caretakers using the same approach and later kept in comparable ways in conspecific packs. Human raising of animals, specifically dogs, has been suggested to lead to the development of positive feelings and behaviors in humans (Nagasawa et al., 2009a; Schneider et al., 2010; Udell and Brubaker, 2016). Moreover, in preparation for standard animal care procedures and scientific tests, the human raised animals at the WSC are trained using positive reinforcement training techniques, i.e., the presentation of a reward (i.e., reinforcement) following a certain behavior which serves to maintain or increase the frequency of that behavior (Vasconcellos et al., 2016). Indeed, the relationship between the trainers and the canids created during positive reinforcement training could represent a scenario where not just the human, but also the animals perceive a person in its environment beneficially and thereby constitute a human-animal bond (Hosey, 2008; Carlstead, 2009; Hosey and Melfi, 2012; Ward and Melfi, 2013; Payne et al., 2015).

Furthermore, interactions between keepers/owners and (captive) animals are thought to differ depending on the individuals involved (Ward and Melfi, 2015; Cimarelli et al., 2016), suggesting that people may form stronger bonds with certain animals than with others. At the WSC, each trainer has varying levels of animal training and professional experience, as well familiarity with each individual compared to that of their fellow trainers resulting in a range of trainer-canid relationships. These established relationships, along with the similar raising and housing of both canid species, allowed us to test which factors influence how the humans and the animals perceive these relationships.

Our aim for this study was twofold. First, we aimed to test the impact of the species and the trainer’s experience, namely their involvement in hand-raising, the overall duration of experience with a specific animal, or the general experience the trainer had with canids on the trainer-canid bonds. To measure these bonds, a human-animal bond survey including questions about the trainers’ bonding with the animals was designed. To test the validity of our questionnaire, we asked not just each trainer to assess their own relationship with a specific animal, but also asked each fellow trainer to assess the human-animal bonds of each of the other trainers. We predicted that trainers would rate their relationship with dogs higher than the relationships they formed with wolves due to dogs being domesticated and thus less dangerous to humans than wolves. Moreover, we predicted that whether a trainer was a hand-raiser of a specific animal or not and the amount of time they spent with the respective animal would increase the feeling of bondedness with that individual.

Second, we set out to test the hypothesis that the established relationship was indeed mutual. To do so, we carried out a greeting test where we investigated the behavior of each animal towards each of the trainers and then tested whether the same factors that dictated how the trainers perceived the relationship with the animals, would also influence how the animals react to the trainers. For example, based on previous studies suggesting that the relationships dogs and wolves form with individual caretakers depend on their past experience with those specific people [e.g., (Gácsi et al., 2005; Kubinyi et al., 2007; Ujfalussy et al., 2017; Wirobski et al., 2021)], we expected that hand-raising a specific animal would result in more affiliative behaviors directed from that individual to the respective human caretaker. Moreover, following the Hypersociability Hypothesis stating that dogs became overall more social towards humans than wolves (Ward and Melfi, 2015; Bentosela et al., 2016; von Holdt et al., 2017), we expected dogs to spend significantly longer in proximity and body contact with humans than wolves. Alternatively, the Canine Cooperation Hypothesis suggests that wolves, being highly social and cooperative towards conspecifics (Range and Virányi, 2015), can also accept humans as social partners if properly socialized and thus unafraid of humans. If this hypothesis is correct, we expected to find no significant species differences between similarly socialized dogs’ and wolves’ behaviors towards humans.

## Materials and methods

### Ethical approval

This study was non-invasive and approved by the institutional ethics and animal welfare committee at the University of Veterinary Medicine Vienna in accordance with Good Scientific Practice guidelines and national legislation (ETK-158/10/2020). Participating trainers signed a consent form allowing the use of their survey answers and their participation in the social interaction test.

### Subjects

Animals tested included 15 adult wolves and 9 adult mixed-breed pack dogs. All animals at the center were hand-raised with conspecifics in peer groups since a young age (within the first 10 days of life) and both species were socialized in the same way, participating in daily care and routines carried out by the trainers of the center (see below). Individual animals were trained with positive reinforcement methods and regularly took part in scientific studies. The packs were moved from one enclosure to another when needed for logistical reasons, e.g., depending on the tests that are to be conducted.

We surveyed 5 trainers and 3 trainer trainees, all female and all within 10–15 years of each other, at the Wolf Science Center, Ernstbrunn, Austria. They had worked at the center for 9.5 months for the newest trainer up to 10.5 years for the most experienced; 4 trainers had participated in the hand-raising of at least one animal. During hand-raising, the trainers bottle-feed and later hand-feed the puppies, sleep with them during the nights (one or two trainers at a time), clean and weigh them in the first few weeks and give them emotional support in stressful situations. At the age of 3–4 weeks, the hand-raisers start to train the puppies simple commands like sit, lie and walk on the leash. Conflict situations are avoided as much as possible and if a conflict arises, the trainers attempt to distract the animal to avoid confrontations. The ‘weaning’ period starts at about 3–4 months of age when the handraisers start to withdraw a bit at a time, e.g., leaving the puppies to sleep alone and then also taking longer and longer breaks during the day. With 5 months they are integrated in packs with adult animals.

### Procedure

#### Human-animal bonds survey

Based on previous work, including the Lexington Attachment to Pets Scale (Hosey et al., 2018) and Carlstead, Paris, and Brown’s survey of keeper-elephant relationships (Carlstead et al., 2019), a new human-animal bonds survey was designed for the present study to assess the human-animal bonds perceived between the trainers and the canids of the center.

The survey was given individually to the trainers who were not aware of the nature of the study during the data collection to reduce the impact this knowledge could have on their answers in the survey or on their behaviors during the interaction test with the canids. The first version of the survey was presented to the trainers for general feedback and was thereafter adjusted to a newer version which was then used for our data collection. The survey was divided into three parts: the first part contains demographic information about the trainer’s experience with the canids with 5 questions (part 1); the second part addresses the individual bonds the trainer has with the animals (self-ratings) with 7 questions (part 2 – “individual human-animal bonds survey”) using a 7-point scale (see Table 1); and the third part includes questions about the bonds other trainers have with the animals (peer-ratings) with 5 questions (part 3 – “trainer human-animal bonds survey”) using a 7-point scale (see Table 2). For both the self-and peer-ratings, each item included the same 7-point scale that ranged from very low to very high, worded to fit the question. The trainers were given the option to not provide a conclusive answer (“I do not know” or “never worked with this animal”). All parts of the survey were completed online *via* smartsurvey.co.uk.

**Table 1:** Survey items for self-ratings of bonds and feelings towards each animal.

Survey question	Item code
Please rate the strength of the bond/positive relationship the animal has with you (from your perspective).	Bond-human perspective
How friendly is each animal to you?	Animal friendliness
Please rate each animal’s intelligence compared to others of this species.	Animal intelligence
Please rate how willing the animal is to approach you.	Animal approach
Please rate how trusting the animal is of you during stressful situations	Animal trust
I like this animal.	Trainer like
Please rate the strength of the bond/positive relationship you have with these animals (from the animal’s perspective).	Bond-animal perspective

**Table 2:** Survey items for peer ratings of animal bonds.

Survey question	Item code
Please rate the strength of the bond/positive relationship your fellow trainers have with each animal (from your perspective).	Bond-human perspective
How friendly is each animal to your fellow trainers?	Animal friendliness
Please rate how willing each animal is to approach your fellow trainers.	Animal intelligence
Please rate how trusting each animal is of your fellow trainers during stressful situations	Animal approach
Please rate the strength of the bond/positive relationship each animal has with your fellow trainers (from the animal’s perspective).	Animal trust

The sample size for the survey consisted of 5 trainers and 3 trainer trainees for the demographic information (part 1) and for the individual human-animal bonds questions (part 2), and 4 trainers and 3 trainer trainees for the trainer human-animal bonds questions (part 3) as one trainer declined to participate in the last part of the survey, which consisted of rating their fellow trainers’ relationships with the canids of the center. The survey included questions on the 15 wolves and 9 dogs initially considered for the sample size. In total, 58 out of 1,308 questions and 307 of 5,880 question of the self and peer ratings, respectively, were answered with “I do not know” or “never worked with this animal.”

#### Social interaction test

Participation in the social interaction tests were voluntary for both the trainers and animals. Each trainer was tested with each canid at the center in a dyadic greeting context. The procedure was based on a previous study conducted at the centre (Wirobski et al., 2021). Prior to each test, the tested animal and their packmates were undisturbed (e.g., no cleaning, feeding or human interaction whatsoever) for a minimum of 30 min. Then, an auxiliary trainer was asked to shift the tested animal’s packmate(s) from the home enclosure to an adjacent enclosure. During this time, the experimenter (MB) placed a camera just outside the enclosure to record the session and then left the area so not to influence the interaction. The animal was given 5 min to acclimate to being alone in the enclosure and then the trainer being tested with the animal walked towards the enclosure door, knelt down, and greeted the animal through the fence for 5 min during which the canid was free to move inside the enclosure and was allowed to end the interaction at any time.

After 5 min, the test was concluded. Trainers were instructed to greet the animal during the 5 min as they wanted depending on their relationship and experience with the animal. The trainers were not given any explicit rules (e.g., how much to call/not call the animal) to not artificially influence the interaction by imposing rules that do not usually apply to the relationship. However, the trainers were asked not to offer or carry food and to take off their trainer jacket before walking to the enclosure to limit animal behavior that might be related to food expectation. The test always took place through the fence for safety.

All dyadic trainer-canid interactions took place with the animals inside their home enclosure, therefore all animals were tested repeatedly in the same enclosure except for two wolves, who were tested in the adjacent new test enclosure of the center for practical reasons, and two dogs, one of whom had to change packs shortly after the beginning of the testing period because of disturbance problems, and the other who we repeated one session with after her pack changed enclosures.

One male wolf and one male dog died due to old age and health problems after we started testing the animals and surveying the trainers. Additionally, one male dog and one female dog were removed from the WSC following medical interventions. Consequently, our final sample size for the interaction tests was comprised of 14 adult wolves (10 M, 4F) and 6 adult mixed-breed pack dogs (2 M, 4 F) (Supplementary results Table S1) as well as 5 trainers and 3 trainer trainees. All 8 trainers were tested once with each of the 20 canids at the center resulting in 160 social interaction sessions of 5 min each (median = 253.80 s, quartiles = 110.89 s). Using focal animal sampling (Altmann, 1974), frequency and durational behaviors were recorded and coded using BORIS program (Behavioral Observation Research Interactive Software).

To code behaviors seen in the social interaction test, we used a modified version of a previously designed ethogram (Wirobski et al., 2021). Specifically, we observed animals for the frequency of self-directed behaviors, licking the hands/face of the trainer, duration of autogrooming and tail wagging, given their uses as indicators of anxiety and social behaviors (Table 3). Additionally, we included an ‘out of sight face’ (OOS face) behavior for adjusting face-related behaviors (yawning, lips licking, licking) by dividing their total occurrences by the time the animal’s face was out of sight, while all the behaviors except for talking were used in proportion of the out of sight (OOS) behavior, so that behaviors and frequencies were adjusted based on how often these behaviors would be observed.

**Table 3:** Social interaction test ethogram.

Behavior	Description	Type
Growling	Animal produces a rough-sounding vocalization, usually low in pitch and loudness.	Duration
Whining	Animal produces an extremely high-pitched “thin” sustained vocalization, usually low in loudness.	Frequency
Yawning	Animal opens their jaws without vocalizing.	Frequency
Body shaking	Animal shakes their body or neck.	Frequency
Lips licking	Animal extrudes their tongue from the mouth and run it over their lips.	Frequency
Scratch	Animal scratches (draws nails or teeth across a body surface) their skin.	Frequency
Autogroom	Animal grooms (licks) their own body.	Frequency
Tail wagging	Animal moves their tail from one side to the other in a repeated way.	Duration
Social contact	Animal is in physical contact with the trainer, who touches them on the side of their body or head.	Duration
Calling	Trainer calls the animal’s name to get their attention.	Frequency
Talking	Trainer talks to the animal.	Duration
Approaching	Animal approaches the fence where the human partner sits in a neutral posture.	Frequency
Leaving	Animal moves away from the fence where the human partner sits (after having interacted).	Frequency
Licking	Animal licks the human partner’s hands or face.	Duration
Proximity to human	Animal stays within one body length of the human partner.	Duration
OOS	Animal’s entire body is out of sight.	Duration
OOS face	Animal’s face is out of sight.	Duration

### Data analysis

All analyses were performed in R (version 3.6.3; R Core Team, 2020).

#### Agreement among raters

All the trainers’ names and answers were dummy coded, and their answers were anonymized. To explore the agreement among the trainers (i.e., the trainers peer-ratings), we used two-way random intraclass correlation coefficients (ICC2k), applied separately for each of the five items, which were derived from the questions of the survey. This was done once considering the ratings of the relationships of the same trainer with different animals, and once considering the ratings of the relationships of the same animal with different trainers.

To estimate the agreement of the trainers’ peer-ratings with the trainers’ self-assessment of their relationship with the animals, we used Kendall’s rank correlation coefficient as we had tied observations and ordinal data. Again, we applied it separately for each item, considering on the one hand the ratings of the relationships of the same trainer with different animals, and on the other hand, the ratings of the relationships of the same animal with different trainers.

The sample for the intraclass correlation (ICC2k) and the Kendall rank correlation coefficient comprised 24 animals and 8 trainers.

#### Data reduction of survey items and social interaction test behaviors

We used Principal Components Analysis (PCA) and subsequent Factor Analysis (FA) to condense the self-assessment of the trainer-animal relationship ratings. We conducted the PCA to identify the number dimensions to retain as the number of Principal Components (PC) with an Eigenvalue > = 1 following the Kaiser-Meyer-Olkin measure of sampling adequacy of 0.88 and also Bartlett’s test of sphericity (McGregor, 1992). Items that were found to have a low correlation with the other items were removed from PCA and FA.

We repeated this strategy with the social interaction behavioral data so to reduce redundancy and aggregate the data. Behaviors that were rare or uncorrelated with the other behaviors were removed from the PCA and FA.

Once a structure was determined, we created separate factor scores for both the survey and behavioral data so to reduce the number of variables included in the generalized mixed effect models (GLMMs).

#### Models to determine the effect of species, sex, and trainer’s past experience with the animals on the human-animal bond

To investigate how the bond between the trainers and the canids of the center differed between species (dog or wolf) and how it was affected by past experience of the trainers with the animals, we fitted four Linear Mixed Models [LMMs (Baayen, 2008)].

In the first model (model 1), to determine which factors might influence the assessment of the human-animal bonds, we predicted the factor “*trainer-animal relationship*” (dependent variable) by the years of experience the trainers had with the canids of the center, whether the trainer was involved in hand-raising the animal, the years of professional work with dogs and wolves, the hours spent per week at the center, the species and the sex of the animal (independent variables).

Next, to investigate whether the animal’s behavior reflects the bond assessment of the human partner or is dependent on other aspects, we ran three additional models (models 2, 3, and 4) each including one of the three factors that were extracted from the behavioral factor analysis as a response variable (dependent variable). As in model 1 we used years of experience the trainers had with the canids of the center, whether the trainer was involved in hand-raising the animal, the years of professional work with dogs and wolves, the hours spent per week at the center, the species, and the sex of the animal as predictor (independent) variables and with the addition of the trainer-animal relationship factor. Doing so allowed us to test the relationship between how the trainer felt about their relationship (the bond strength) and the animal’s behavior towards them.

Into all four models we included random intercept effects of the ID of the animal and the trainer to avoid pseudo-replication. To avoid overconfident models and keep type I error rate at the nominal level of 0.05, we included random slopes (Schielzeth and Forstmeier, 2009; Barr et al., 2013) that model potential variation among the individual animals and trainers with regard to the effect of the fixed effects predictors. More specifically we included all theoretically identifiable random slopes, namely those of involvement in hand-raising, hours per week working at the WSC, the relationship factor, the years of experience with the specific individual, and the number of years of professional work with canids within animal ID, and of the relationship factor, hours per week working at the WSC, animal sex, species, the years of experience with the specific individual, and the number of years of professional work with canids within trainer ID into models 1, 2, and 3. Model 4 had an almost identical random effects structure, but lacked the random slopes of the relationship factor and also that of the hours per week working at the WSC within trainer ID. Originally, we also included parameters estimating the correlations among random intercepts and slopes. However, in all models, several of them appeared to be unidentifiable as indicated by absolute correlation parameters close to 1 (Matuschek et al., 2017), which was probably caused by the models getting very complex given the available sample size (see below). Hence, we excluded those correlation parameters.

As an overall test of the effects of the fixed effects test predictors and to avoid cryptic multiple testing (Schielzeth and Forstmeier, 2009), we compared each full model with a respective null model lacking them but being otherwise identical. To test the significance of individual test predictors we dropped them, one at a time, and compared the resulting reduced with the respective full model (Barr et al., 2013). For all these model comparisons we used likelihood ratio tests (Dobson, 2002).

We fitted the models in R [version 3.6.3 (R Core Team, 2020)] using the function lmer of the package lme4 [version 1.1-21, (Bates et al., 2014)]. Prior to fitting the models, we log-transformed the number of years of experience with the specific individual and the number of years of professional work with canids. Subsequently we z-transformed all covariates (i.e., quantitative predictors) to a mean of zero and a standard deviation of one to ease model convergence. Before including involvement in hand-raising as a random slope, we manually dummy coded and then centered it. To achieve roughly normally distributed and homogeneous residuals (verified by visual inspection of a qq-plot of the residuals and residuals plotted against fitted values), we square root transformed the three behavioral factors (after subtracting their respective minimum). All models were fitted using maximum likelihood. We estimated model stability by dropping animals and trainers from the data, one at a time, fitting the full model to the obtained subsets, and finally comparing the model estimates derived for these subsets with those obtained from the full data set. This revealed the models to be of moderate to good stability in the fixed effects part. We estimated confidence intervals of model estimates and fitted values by means of parametric bootstraps (*N* = 1,000 bootstraps) which we conducted using the function bootMer of the package lme4.

The sample for all four models comprised a total of 160 observations involving 20 animals and 8 trainers.

## Results

### Agreement among raters: Intraclass correlations and Kendall’s Tau

Median peer-ratings’ ICC2k of the ratings of a given subject’s relationships with different trainers ranged from 0.74 (*Animal approach*) to 0.93 (*Bond-animal perspectiv*e). Similarly, the ICC2k of the raters’ agreement about a given trainer’s relationships with different subjects ranged from 0.86 (*Animal approach*) to ICC = 0.93 (*Animal friendliness*). As for the Kendall rank correlation, median peer-ratings’ Kendall’s tau of a given subject’s relationships with different trainers ranged from 0.457 (*Animal approach*) to 0.722 (*Bond-animal perspectiv*e), whereas for the trainer’s relationships with different subjects, it ranged from 0.53 (*Animal friendliness*) to 0.64 (*Bond-animal perspective*). These results suggest that trainers agree in the assessments of the relationships they form with the animals.

### Principal components analysis and factor analysis of the surveys

When including all six items from the trainers’ ratings of their own relationships with the animals (*Animal approach*, *Bond-animal perspective*, *Bond-human perspective*, *Animal friendliness*, *Animal trust*, and *Trainer like*), using the PCA we found a single component structure was suggested with an Eigenvalue ≥1, but “Trainer like” did not load strongly on the first PC, and also not strongly on the factor revealed by an FA comprised of one factor (and also not strongly on any of the first two factors when we used an FA with two factors). Because of this and also because ‘Trainer like’ was not strongly correlated with any of the other items, we excluded it from the PCA/FA. The ‘intelligence’ code was also excluded from the analysis because it was not correlated with the ratings of relationships between the trainers and the animals. A PCA on the five remaining items suggested a single component structure with an Eigenvalue >1 and the single factor (representing the relationship variables), hereafter referred to as the “relationship factor,” extracted by a subsequent FA explained 82.66% of the total variance (Eigenvalue: 4.13) on which all five items loaded highly (Table 4).

**Table 4:** Human-animal bonds items factor structure.

Item	Loading
Animal approach	0.852
Bond-animal perspective	0.961
Bond-human perspective	0.891
Animal friendliness	0.895
Animal trust	0.942
Eigenvalue	4.133
Percent variance explained	0.827

### Principal components analysis and factor analysis of the behavioral data

For the behavioral data, eight behaviors were removed from the PCA, namely: whining, body shaking, scratch, autogroom, growling, talking, calling, and yawing, mostly due to rarity and/or poorly correlated with the other behaviors and contributed little to any of the PC/factors constructed with initial PCAs/FAs including them. With the remaining seven behaviors, a PCA was clearly justified (Kaiser-Meyer-Olkin measure of sampling adequacy: 0.686; Bartlett’s test of sphericity: c2 = 1318.1, df = 21, *p* < 0.001), which suggested a three components structure with an Eigenvalue >1. The three factors constructed using the subsequent FA explained a total of 82.1% of the total variance in the variables. Factor I correlated mainly with the proportion time the animal approached and left the trainer; Factor II mainly with the proportion time the animal was wagging its tail, the proportion of time they licked the trainer’s hand or face and the number of times (per minute) they licked their lips; and Factor III mainly with the proportion of time in physical contact with the trainer and the proportion of time the animal stayed in proximity to the trainer (see Table 5).

**Table 5:** Social interaction behavioral factor structure.

Behavior	Factor I	Factor II	Factor III
Proportion of time approaching	**0.986**	−0.115	−0.102
Proportion of time leaving	**0.983**	−0.124	−0.122
Proportion of time in physical contact	−0.393	0.364	**0.739**
Proportion of time staying with human	−0.009	0.227	**0.971**
Portion of time wagging tail	−0.119	**0.795**	0.21
Licking per minute	−0.045	**0.667**	0.11
Lips licking per minute	−0.153	**0.833**	0.253
Eigenvalue	2.133	1.982	1.635
Percent variance explained	0.305	0.283	0.234

### Factors influencing the assessment of the human-animal bond

**Figure 1:**
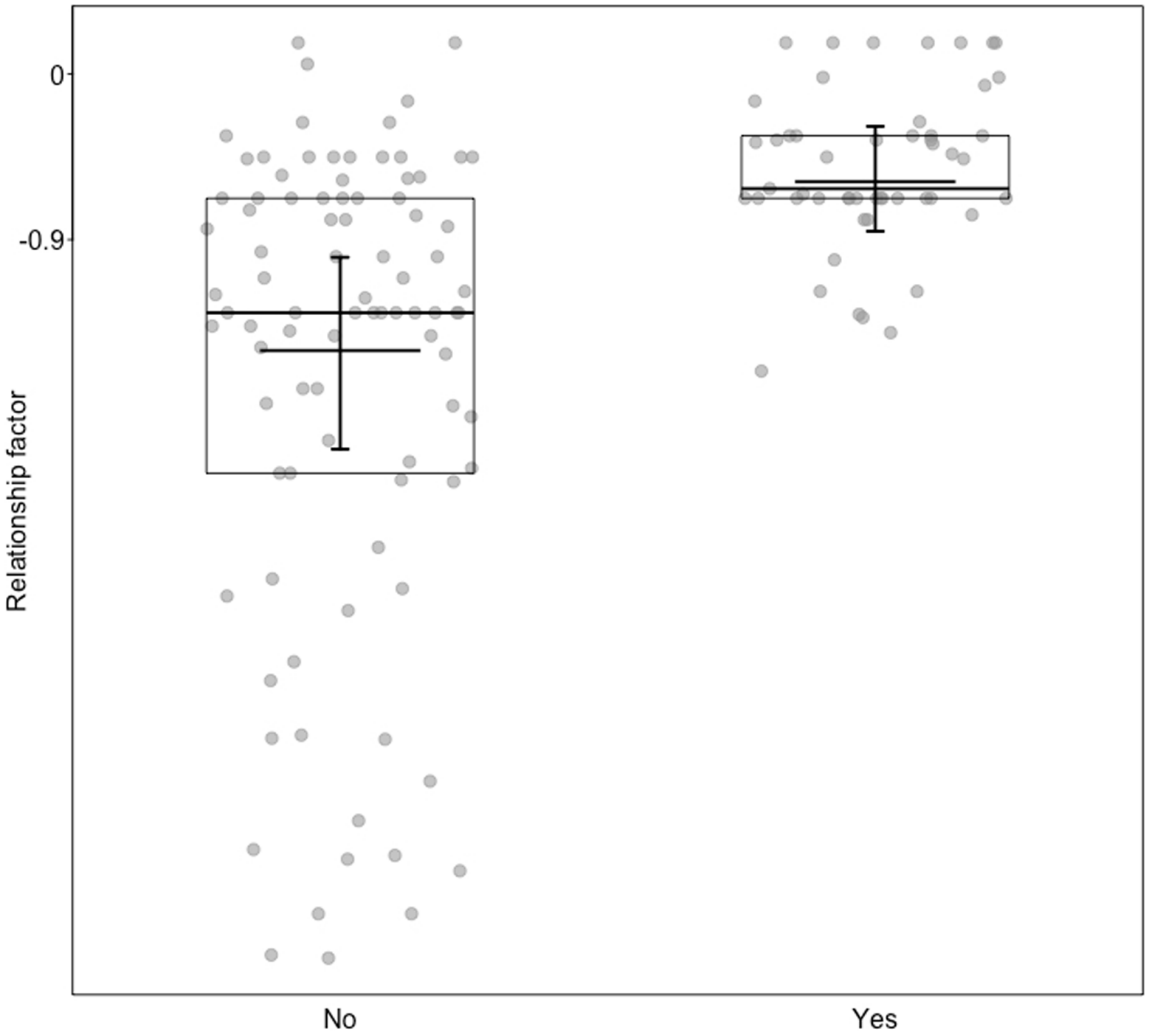
Relationship factor (*y*-axis), as a function of if the trainer was hand-raiser (yes) or not (no). Dots show the individual data, horizontal lines with boxes medians and quartiles, and the short horizontal lines with error bars the fitted model and its confidence limits with all other predictors centered to a mean of zero. The relationship factor was comprised of ratings of how often the animal approaches, the strength of the bond from the trainer’s perspective, the strength of the bond from the animal’s perceived perspective, how friendly the animal is to the trainer, and how much the animal trusts the trainer.

In the first model with the relationship factor as a response, which was comprised of the items from the bonds surveys, we found a significant full-null model comparison (c2 = 16.712, df = 5, *p* = 0.005). If a trainer had been a hand-raiser for a specific animal, she assessed her relationship with the animal higher than when not [b [95*%* CI] *=* 0.79 [0.26, 1.31]*, p <* 0.01; Figure 1; Supplementary results Table S6]. A trainers’ years of professional work, experience with the individual animals, and hours working at the WSC had no effect, nor did the animals’ species or sex (Supplementary results Table S6).

### Factors influencing the behavior of the animals

For model 2 predicting the behavioral factor I, which was mainly comprised of proportion of time approaching and leaving, we found the full-null model comparison not to be significant (c2 = 3.59, df = 6, *p* = 0.732). Correspondingly, there were no significant effects of the relationship factor, the years of experience the trainers had with the individuals, whether the trainer’s hand-raised the animals, their professional years of experience with wolves and dogs, the hours the trainers spend at the center per week, and the species on the behavioral factor I (Supplementary results Table S7).

**Figure 2:**
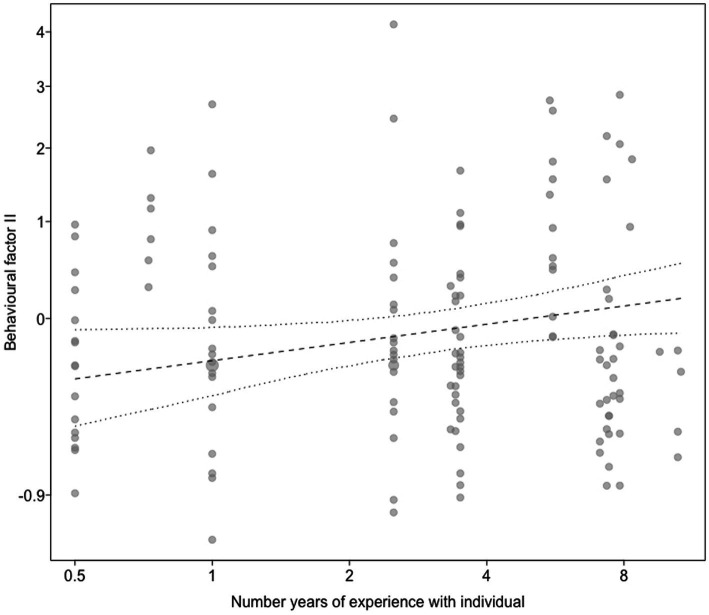
Behavioral factor II (*y*-axis) as a function of the number of years of experience with the individual (*x*-axis). Dots show the individual data, the straight line represents the predictive mean of the model, and the dashed on dotted lines the fitted model and its 95% confidence limits with all other predictors centered to a mean of zero. Behavioral factor II was mainly correlated with proportion time tail wagging, proportion time licking trainer’s hand or face, and numbers of lips licking.

**Figure 3:**
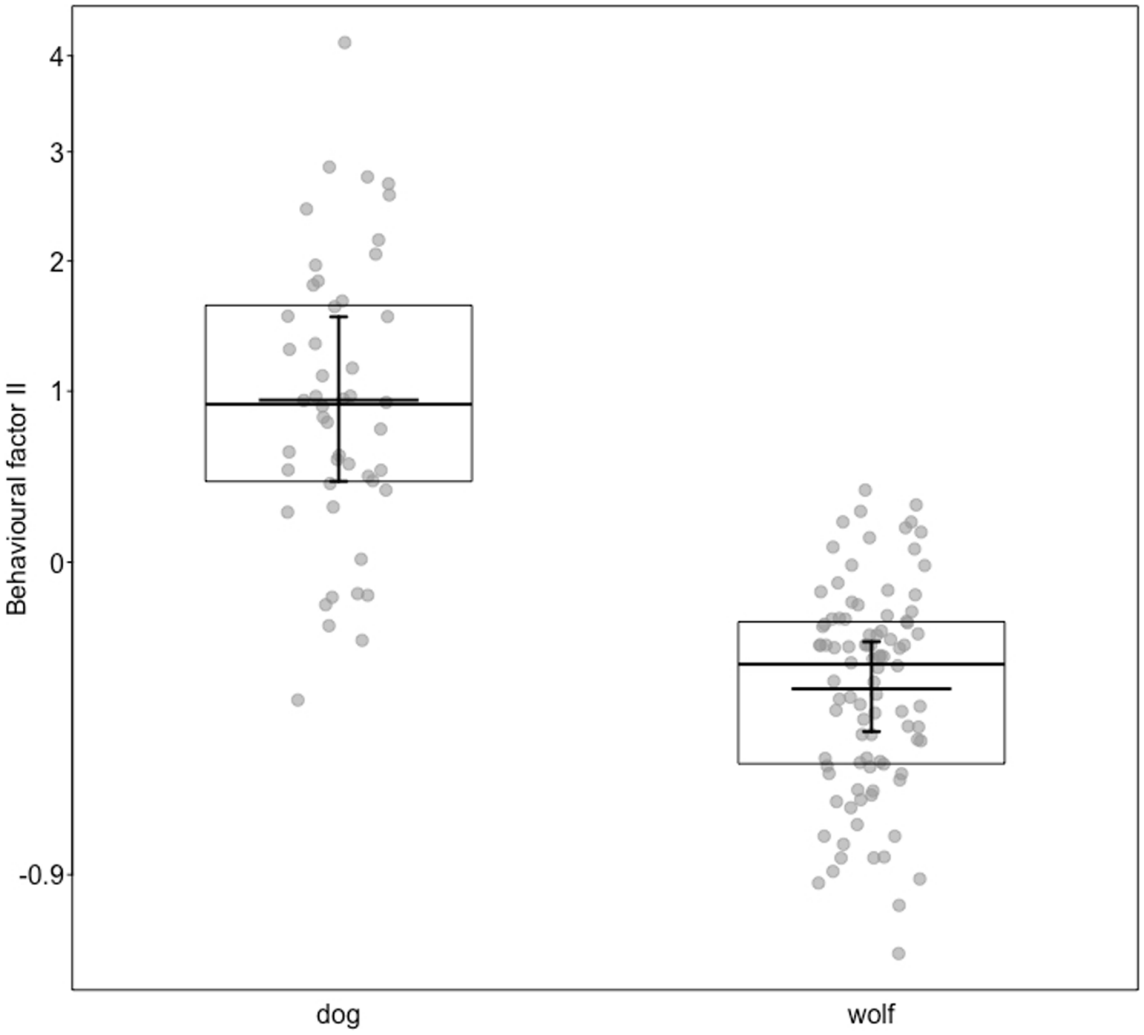
Behavioral factor II (*y*-axis), separately for dogs and wolves. Dots show the individual data, horizontal lines with boxes medians and quartiles, and the short horizontal lines with error bars the fitted model and its 95% confidence limits with all other predictors centered to a mean of zero. Behavioral factor II was mainly correlated with proportion time tail wagging, proportion time licking trainer’s hand or face, and numbers of lips licking.

For model 3 predicting the behavioral factor II as a response, which was mainly comprised of the proportion of time tail wagging, licking the trainer’s hand or face, and the frequency of lips licking, we found a clearly significant full-null model comparison (c2 = 27.44, df = 6, *p* < 0.001). More specifically, the more years of experience the trainer had with an animal, the higher the behavioral factor II (i.e., the more it wagged its tail, licked the trainer’s hand or face, and demonstrated lips licking; b [95*%* CI] *=* 0.11 [0.01, 1.21]*, p <* 0.041; Supplementary results Table S8; Figure 2. Moreover, dogs were more likely to show these behaviors than wolves; b [95*%* CI] *= −*0.72 [−0.94, −0.46]*, p <* 0.001; Supplementary results Table S8; Figure 3). On the other hand, the relationship factor, the fact that the trainer was involved in an animal’s hand-raising, the years of professional work with dogs and wolves, the hours spent per week at the center, and the sex of the animal were not significant (Supplementary results Table S8).

**Figure 4:**
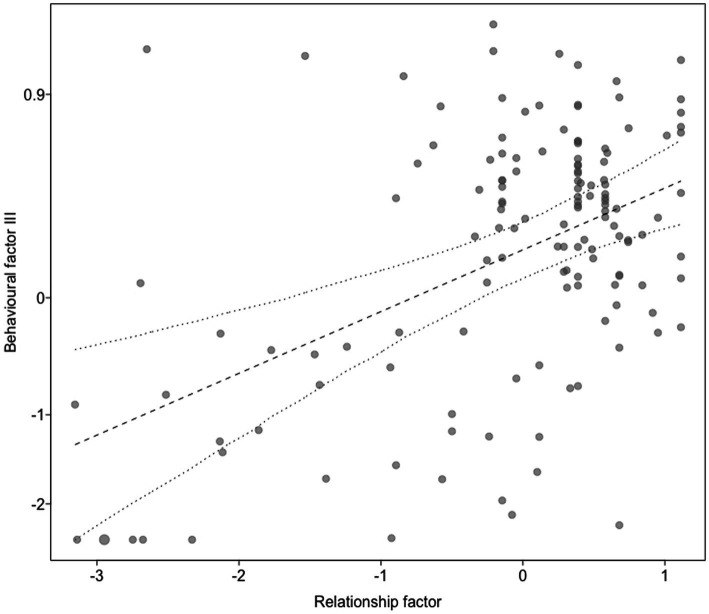
Behavioral factor III (*y*-axis) as a function of the relationship factor (*x*-axis). Dots show the individual data, the straight line represents the predictive mean of the model, and the dashed on dotted lines the fitted model and its 95% confidence limits with all other predictors centered to a mean of zero. Behavioral factor III was mainly correlated with proportion time petting and proportion time staying with human.

**Figure 5:**
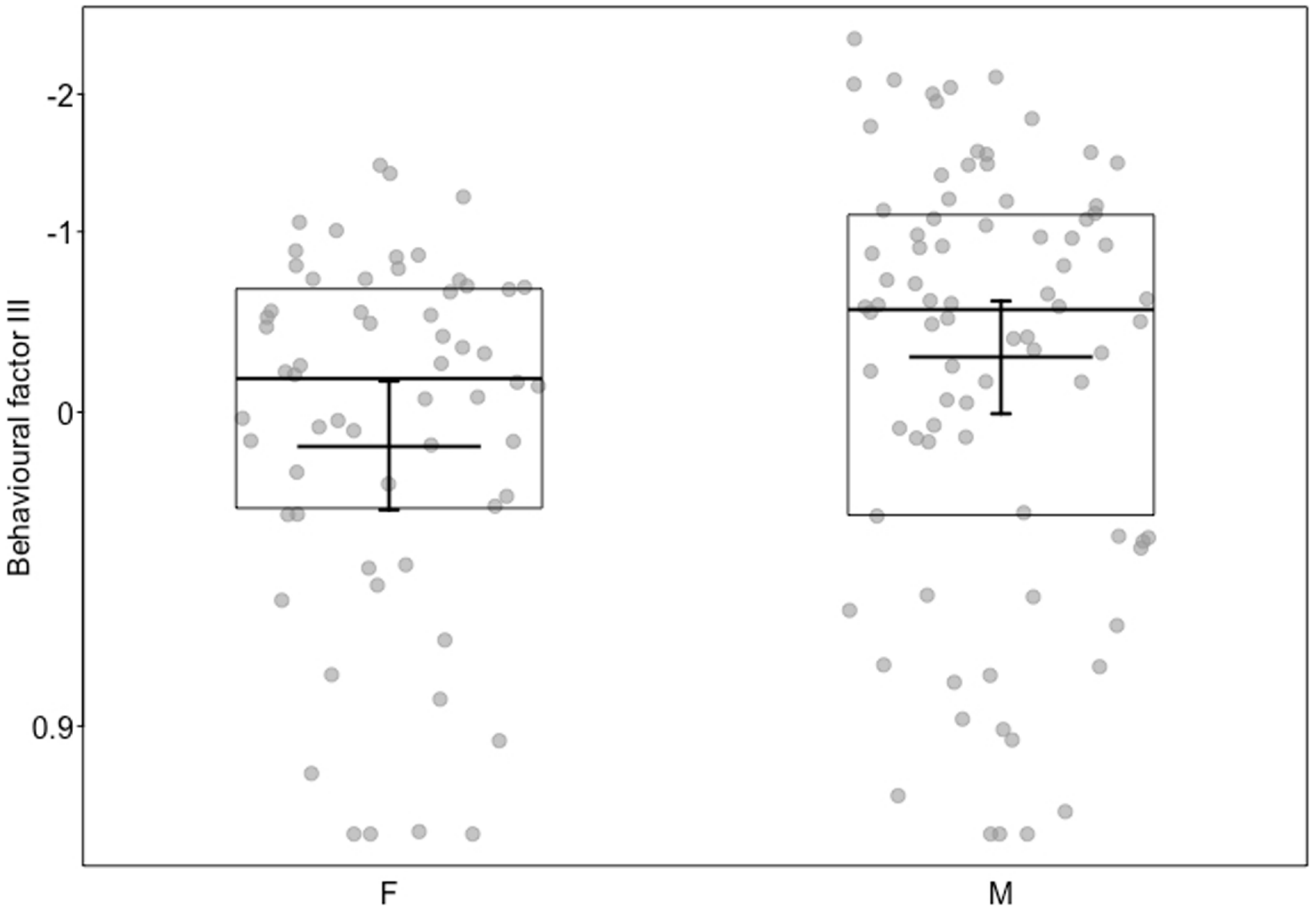
Behavioral factor III (*y*-axis) as a function of sex (*x*-axis; F = female, M = Male). Dots show the individual data, horizontal lines with boxes medians and quartiles, and the short horizontal lines with error bars the fitted model and its confidence limits with all other predictors centered to a mean of zero. Behavioral factor III was mainly correlated with proportion time petting and proportion time staying with human.

For the final model with the behavioral factor III as a response, which was mainly comprised of proportion of time in physical contact and the proportion of time staying with human (proximity), we also found a significant full-null model comparison (c2 = 17.19, df = 6, *p* = 0.009) and both the relationship factor (b [95% CI] = 0.23 [0.13, 0.33], *p* < 0.001) and the sex of the animal were significant (b [95% CI] = 0.21 [0.00, 0.41], *p* < 0.044; Supplementary results Table S9). The higher the trainers rated their relationship with the animal, the more time the animal spent in proximity to and in physical contact with the trainers (Figure 4). Additionally, male individuals stayed closer and in more contact with the trainer compared to female individuals (Figure 5). The years of experience the trainers had with the individuals, their involvement in hand-raising them, the years of professional work with dogs and wolves, the hours of work spent per week at the center as well as the species were not significant.

## Discussion

In the present study, we set out to (1) investigate how species, hand-raising and experience influences the perception of the bond animal trainers form with their canid partners and (2) test whether the factors influencing the trainers’ perception of the relationship with the animals would also predict the animals’ reactions to the trainers, thereby testing also predictions of the Hypersociability and the Canine Cooperation hypotheses. Thus, we attempted to elucidate the human-canine relationship considering both the human as well as the animals’ perspective. In regard to our first aim, we found high agreement between self-and peer ratings of the relationships the animal trainers formed with the wolves and dogs at the Wolf Science Center, suggesting the validity of our questionnaire. The self-ratings of the various relationship characteristics loaded onto a single ‘relationship factor’, indicating they all belong to a single bond construct. Interestingly, the relationship factor characterizing a specific human-animal bond was mainly predicted by whether the trainer had been a hand-raiser for the particular animal rather than the duration of experience the trainer had with the animal or whether it was a dog or a wolf, suggesting that this early, very intense time with the animal was decisive on how the bond was perceived. When testing how the animals perceived their relationships with the individual trainers in a greeting test (second aim), we found that their behaviors loaded on three factors: Factor I included approach and leaving, Factor II lips-licking, licking trainer’s hand or face and tail-wagging and Factor III contact and proximity. When analyzing whether the ‘relationship factor’, species, and/or experience predicted the behaviors of the animal, we found that the humans’ assessment of their relationship with the specific animal as well as sex of the animal predicted the proximity/contact of the animal rather than species, thus refuting the hypersociability hypothesis. Tail-wagging, lips-licking, and hand-licking on the other side was predicted by species and experience with the trainer. To our knowledge, this is the first study to investigate human-animal bonds of animal trainers and similarly raised and socialized adult wolves and dogs using a bi-directional approach.

The first part of the study consisted of creating and validating a new human-animal bond survey. In order to ensure unambiguous questions, we asked the trainers for some feedback on a first version of the questionnaire. While this might have allowed the trainers to anticipate questions in the main survey, we do not think it influenced their final answers in any way that could be problematic for our results, since, even if they thought a bit about the relationship they formed with the animals before filling our final survey, it would have made their answers just more accurate.

Overall, trainers agreed on the bond assessments in the self-and peer-ratings, suggesting that our survey is a good measure for evaluating the bonds that the trainers establish with the animals. As there are no similar studies on self-and peer-ratings in human-animal bonds, we compared our results to personality studies. Usually, interrater reliability in personality questionnaires range from 0.50 to 0.80 (McCrae and Costa, 1987; Gosling and Vazire, 2002; Ley et al., 2009; Salonen et al., 2021), suggesting our interrater reliability estimates were mostly very good and in line with previous studies. The WSC working context requires staff to be responsive to the animals’ behaviors and personality, while working together with the other trainers and witnessing their involvement and experience with the canids, which are constantly evolving depending on the interactions taking place on a daily basis. In that respect, the trainers may be prone to perceive and evaluate the human-animal bonds in a similar manner. However, this is similar to most studies assessing interrater reliability of questionnaires related to dogs’ personality, since they usually use assessments of different family members to define interobserver reliability (McCrae and Costa, 1987; Gosling and Vazire, 2002; Ley et al., 2009; Salonen et al., 2021). Alternatively, it is possible that, despite very clear instructions not to do so, the trainers did communicate with each other about the questionnaire and formed a joined opinion about the relationships the animals form with each other. While we cannot completely rule this out, we think it is very unlikely for two reasons. First, our trainers are professionals that understand the importance of science and thus adhere to the instructions we give them. Second, discussing the relationship each of the 8 trainers has with each of the 24 animals and finding an agreement, would have taken up an enormous amount of time, which our trainers just do not have during their daily work.

When analyzing the self-ratings of the trainers, the factor analysis revealed a single ‘relationship’ factor, that explained almost 83% of the variance. Perhaps not surprisingly, trainers involved in hand-raising an animal rated their relationship with the animal as better than when not involved in the hand-raising. Hand-raising the canids at the WSC requires a high commitment from the trainers involved, which comprises of taking care of the young animals extensively and on a daily basis, thus creating a close relationship throughout the development of the animal, which likely helps creating and/or perceiving a bond significantly stronger than if not involved in the hand-raising. Surprisingly, the amount of time the trainers have spent in interactions with the animals did not influence this judgement, which is in contrast with studies showing that HAB were more likely to be reported among zoo keepers who had daily visual contact, fed and talked to the animals they care for than those that did not have as much contact (Hosey and Melfi, 2012). It appears that trainers who helped hand-raise the animals have acquired a certain feeling of bondedness and trust with specific animals that is not acquired by frequent interactions. This is in line with our personal observations, that it sometimes may take years to establish a trustful relationship, especially with the wolves, if the person was not involved in the hand-raising, and that with some animals, even years are not enough to allow for safe and relaxed interactions with certain people. Interestingly, the animal’s behavior does not confirm the importance of hand-raising for their interaction with the trainer (see below).

Interesting, the relationship factor was not predicted by species, suggesting that trainers did not feel more bonded with one species than the other. These results were somewhat surprising since, as an effect of domestication, we predicted that humans might be more open to build up a trustful bond with dogs rather than wolves. A potential explanation for this result might be due to using explicit (questionnaire) rather than implicit methods to investigate the attitude of the trainers to the animals. Indeed, whereas implicit processes are thought to be evoked automatically by the stimulus, are robust and run to completion without direct monitoring, potentially occurring without insight and awareness (Gyurak et al., 2011), explicit processes are slower, the result of the involvement of reflective processes and considered to be based on domain-general mechanisms subject to conscious control (Evans, 2008). Given the goal of the research center to raise and keep wolves and dogs ensuring that they have the same experiences and receive the same treatment, it is possible that the trainer’s self-reported emotions and attitudes towards the two species do not necessarily match their more visceral, implicit reactions. This would be in line with extensive social-cognitive research that revealed significant discrepancies between self-report methods (e.g., explicit methods) and more direct measures (e.g., implicit association test) thought to tap into the more implicit reaction to specific stimuli (Hofmann et al., 2005). Here, further studies are needed to test whether explicit and implicit attitudes align. On the other hand, the extensive, long-term relationship the trainers and the animals engage in at the center could indeed lead to them forming very similar, emotional relationships with the animals irrespective of species (Hosey and Melfi, 2012; Hosey et al., 2018). Of course, the trainers at the Wolf Science Center are a unique group of people so that the results might not be generalizable to the general public. However, to test if people can form similar relationships with wolves and dogs, it is necessary that they have similar experiences with both species. Thus, while our sample size is small and very special, it will be hard to test a larger number of people where this assumption is met.

It could also be argued that the number of trainers used in the surveys’ principal components analysis and factor analysis is too small for a proper analysis given the number of variables leading to overfitting. However, in PCA and FA the sample size is not the number of observers but the number of observations that goes into the analysis (personal communication with psychometrician). For our study, we have 192 observations (8 trainers rating 24 animals equaling 192, on 5 items); this equates to a ratio of 38:1 for observations to items, which makes our structure appropriate (Wolf et al., 2013). Moreover, since we have no missing data and each variable highly loads on a single factor, our data meet the requirements considered solid (Costello and Osborne, 2005; https://www.theanalysisfactor.com/sample-size-needed-for-factor-analysis/). However, we do acknowledge that we might have a potential problem with autocorrelation, since we used the same observers throughout, which could influence our error terms.

In the second part of the study, we assessed the animal’s reactions towards the trainers in a simple greeting test through the fence. We asked whether the assessment of the bond by the human (‘Relationship factor’) also predicted the behavior of the animal, in other words whether they perceived the bond similarly as the trainers.

The relationship factor did predict how much time the animals spent in proximity/contact with the trainer, suggesting that there is a mutual agreement on the strength of the bond the animal and trainer share. Indeed, each animal was free to move inside the enclosure and enter or leave the interaction at any time, while the trainer would stay next to the enclosure door. Thus, the choice of coming into proximity to the trainer was made by the animal, which demonstrates a certain tolerance and trust for staying in proximity and being petted.

Interestingly, and contrary to our experience-based hypothesis, both ‘the years of experience with the animal’ and ‘the involvement in hand raising’ variables were not significant in enlisting the proximity and contact behaviors, suggesting that, at least from the animals’ perspective, there is some other aspect of the relationship that drives this response. Possibly, canids are more comfortable being in proximity and body contact with trainers that rate their bond with an animal strongly and thus likely are relaxed and confident in proximity of the animal. Additionally, the trainer could have rated the bond according to the usual behaviors she experiences with the animal on a day-to-day basis in combination with the idea that hand-raising is an important aspect of building up a relationship. Since bonds are two-sided, it will be difficult to differentiate between cause and effect; however, implicit measures such as an implicit association test could help to at least partly disentangle the influence of knowledge of the goal of the center on these assessments.

Males, both wolves and dogs, spent more time in proximity and contact with the trainers than females in our study. Scandurra et al. (2018) suggested that male and female dogs tend to show different levels of interspecific sociability depending on the context: male dogs can display more social contact than females regarding their engagement in dog-human social play, while the opposite has been reported in cooperative contexts when the animals were trying to solve a problem. Knowing this, it’s possible that in a different context, such as during a cooperative task, the female WSC animals may have shown more sociability. Nonetheless, more data is needed to better explore sex-differences in canids regarding their interactions with humans given that our sample size is rather small.

In contrast to other greeting/proximity studies [e.g., (Bentosela et al., 2016)], we did not find any significant differences in proximity seeking between wolves and dogs. Accordingly, our data support the prediction of the Canine Cooperation Hypothesis that wolves, if properly socialized, can accept humans as social partners (Range and Virányi, 2015). However, we tested the animals only with people with whom they have a very close relationship, which might explain the lack of a differences [see also (Hall et al., 2015; Wheat et al., 2020; Wirobski et al., 2021)]. The hypersociability hypothesis predicts that dogs have an increased social interest compared to wolves [“exaggerated motivation to seek social contact,” (von Holdt et al., 2017)]. Unfortunately, it is unclear from the original publication, if this prediction only applies to people the animals do not know or also to familiar people (Bentosela et al., 2016; von Holdt et al., 2017), making it difficult to draw a firm conclusion.

Results from our third model show that the more years of experience the trainer had with an animal at the center, the more the animal wagged its tail, licked the trainer’s hand or face, and demonstrated lip-licking. Tail-wagging in dogs has been referred to as a contact-seeking and communicative behavior (Norling et al., 2012; Rehn et al., 2014) and has shown to be displayed more towards the dog’s owner compared to strangers (Prato-Previde et al., 2003; Kuhne et al., 2012; Norling et al., 2012). Accordingly, the higher frequency of tail-wagging with more experienced trainers might be indicative of the affiliative relationship the animals have with the trainers. However, whether tail-wagging is indicative of positive or negative arousal seems to depend on the direction of the movement of the tail in the horizontal plane with tail wagging to the left being associated with positive arousal (Quaranta et al., 2007). Unfortunately, we did not measure the tail-wagging direction in this study.

Moreover, lip-licking is often considered as a stress signal (Beerda et al., 1998; Csoltova et al., 2017). However, both lip-licking and licking the face of another individual is an important component of canid greeting behavior that is performed by the submissive towards the more dominant individual (Fox, 1970). Similar, in animal-human interactions, these signals may primarily serve as communicative cues during greeting of familiar individuals (Kuhne et al., 2012; Shiverdecker et al., 2013; Rehn et al., 2014), increasing the attention from the human (Norling et al., 2012; Rehn et al., 2013) or indicating higher emotional arousal (Rehn et al., 2013). They often occur in social situations perceived as mildly threatening and might function to avoid conflicts (Firnkes et al., 2017). Given that we set up a greeting situation, it is possible that the display of submissive signals is stronger, the clearer and better the relationship is between animal and trainer, e.g., the more experience they have with each other. This is especially likely, since animal-human interactions at the Wolf Science Center are mainly occurring in formal training situations, where the trainer asks for certain behaviors to be carried out by the animal in exchange for a treat, i.e., ‘formal’ leader, follower interactions. Additionally, the higher frequency of these communicative cues in dogs compared to wolves could be indicative of higher submissiveness of the dogs towards the trainer compared to the wolves. This interpretation is in accordance with a previous study that was carried out at the Wolf Science Center (Wirobski et al., 2021) and lends tentative support to the notion that dogs were selected for increased submissive inclinations, ultimately resulting in more compliant cooperation partners for humans [Deferential Behavior hypothesis (Range et al., 2019)].

Approaching/leaving was not influenced by any of the factors we tested. Studies with farm animals have explored approach/avoidance behaviors towards humans as part of the human-animal bonds research (Hosey and Melfi, 2014); however, approach behavior can be due to several conflicting motivations, for instance the investigation of novel objects or unfamiliar humans as well as expectations of the approaching human (Waiblinger et al., 2003). Similarly, the canids in our study might have different motivations when approaching or leaving the trainers, which can be influenced by environmental factors; for instance, while coding the videos we noticed that sometimes the animals left the trainers after being distracted by some external factor (e.g., noise). These behaviors may thus have been displayed for different reasons not directly linked to the relationship the canids maintain with the trainers, which could explain why we did not find any significant results for this analysis.

To conclude, our study revealed that the perceived bonds the trainers form with the wolves and dogs at the Wolf Science Center are mainly predicted by having been a hand-raiser or not and not by species. The animals reciprocate the perception of the trainers in that they stay more in proximity with trainers that assess their bond as good, regardless of if they were hand-raisers or not. This suggests that some other unmeasured variable may be influencing the strength of the bond from the animals’ perspective. While we investigated the canine-human relationship in this study, humans form also bonds with other domesticated and non-domesticated species and the animals react to these bonds (Hosey, 2008; Carlstead, 2009; Hosey and Melfi, 2012; Ward and Melfi, 2013; Payne et al., 2015). Further investigation of these relationships might reveal similar patterns.

## Data availability statement

The original contributions presented in the study are included in the article/Supplementary results material, further inquiries can be directed to the corresponding authors.

## Ethics statement

This study was non-invasive and approved by the institutional ethics and animal welfare committee at the University of Veterinary Medicine Vienna in accordance with Good Scientific Practice guidelines and national legislation (ETK-158/10/2020). Participating trainers signed a consent form allowing the use of their survey answers and their participation in the social interaction test.

## Author contributions

FR acquired the funding. LR, MB, SW, and FR designed the study. MB conducted the experiments. MB and LR managed the data. MB, LR, and FR took care of the data analysis and its interpretation and wrote the manuscript. The manuscript was written by MB, FR, and LR. SW commented on the MS. All authors contributed to the article and approved the submitted version.

## Funding

The research was funded by the Austrian Science Funds (Project number: P34675-G and 30704-B29) to FR.

## Conflict of interest

The authors declare that the research was conducted in the absence of any commercial or financial relationships that could be construed as a potential conflict of interest.

## Publisher’s note

All claims expressed in this article are solely those of the authors and do not necessarily represent those of their affiliated organizations, or those of the publisher, the editors and the reviewers. Any product that may be evaluated in this article, or claim that may be made by its manufacturer, is not guaranteed or endorsed by the publisher.
