# Supplementary material for: Bonded by nature: Humans form equally strong and reciprocated bonds with similar raised dogs and wolves
Source: Front Psychol. 2023 Jan 4;13:1044940. doi: 10.3389/fpsyg.2022.1044940 (PMC9846132; doi:10.3389/fpsyg.2022.1044940)
Supplement: Supplementary file 6 [file Table_2.docx]

**Supplementary Material for ‘Bonded by nature: Humans form equally strong and reciprocated bonds with similar raised dogs and wolves’**

Burkhard, M. ^1^, Range, F.^1*^, Ward, S. J.^2^, and Robinson, L.M.^1,3*^

Please find below the tables for the outputs for the various models:

Table S6 Results of the Linear Mixed Model with the relationship factor as a response

| Term | Estimate | SE | Lower Cl | Upper Cl | df | P |  | |  |  |  |  |  |  |  |  |  |  |  |  |
| --- | --- | --- | --- | --- | --- | --- | --- | --- | --- | --- | --- | --- | --- | --- | --- | --- | --- | --- | --- | --- |
| Intercept | -0.23 | 0.25 | -0.73 | 0.25 |  | (1) |  | |  |  |  |  |  |  |  |  |  |  |  |  |
| Years of experience with individual(2) | 0.16 | 0.09 | -0.04 | 0.36 | 1.00 | 0.15 |  | |  |  |  |  |  |  |  |  |  |  |  |  |
| Hand-raiser (yes)(3) | 0.79 | 0.27 | 0.26 | 1.31 | 1.00 | **0.010** |  | |  |  |  |  |  |  |  |  |  |  |  |  |
| Years of professional work(4) | 0.02 | 0.13 | -0.27 | 0.30 | 1.00 | 0.88 |  | |  |  |  |  |  |  |  |  |  |  |  |  |
| Hours per week at WSC(5) | -0.08 | 0.10 | -0.27 | 0.12 | 1.00 | 0.47 |  | |  |  |  |  |  |  |  |  |  |  |  |  |
| Species wolf(6) | -0.07 | 0.13 | -0.36 | 0.21 | 1.00 | 0.63 |  | |  |  |  |  |  |  |  |  |  |  |  |  |
| Sex male(7) | -0.07 | 0.12 | -0.34 | 0.18 | 1.00 | 0,59 |  | |  |  |  |  |  |  |  |  |  |  |  |  |
| (1) not indicated because of having a very limited interpretation | | | | | | | |  | | |  | | |  | |  | |  | |  |
| (2) log and then z-transformed to mean of zero and a standard deviation (sd) of one; mean and sd of the original log-transformed variable were 0.985 and 0.943 | | | | | | | |  |  |  |  |  |  |  |  |  |  |  |  |  |
| (3) dummy coded with no being the reference category | | | | | | | |  | |  | | |  | |  | |  | |  | |
| (4) log and then z-transformed to mean of zero and a standard deviation (sd) of one; mean and sd of the original log-transformed variable were 1.355 and 0.983, respectively | | | | | | | |  |  |  |  |  |  |  |  |  |  |  |  |  |
| (5) z-transformed to mean of zero and a standard deviation (sd) of one; mean and sd of the original variable were 35.915 and 4.559, respectively | | | | | | | |  | | | |  |  |  |  |  |  |  |  |  |
| (6) dummy coded with dog being the reference category | | | | | | | |  | |  | | |  | |  | |  | |  | |
| (7) dummy coded with F being the reference category | | | | | | | |  | |  | | |  | |  | |  | |  | |

Table S7. Results of the Linear Mixed Model with the behavioral factor I as a response

| Term | Estimate | SE | Lower Cl | Upper Cl | df | P |  |  |  |  |
| --- | --- | --- | --- | --- | --- | --- | --- | --- | --- | --- |
| Intercept | 0.84 | 0.11 | 0.59 | 1.06 |  | (1) |  |  |  |  |
| Relationship factor(2) | 0.09 | 0.07 | -0.05 | 0.24 | 1 | 0.23 |  |  |  |  |
| Years of experience with individual(2) | -0.02 | 0.05 | -0.14 | 0.09 | 1 | 0.77 |  |  |  |  |
| Hand-raiser (yes) | -0.03 | 0.07 | -0.19 | 0.14 | 1 | 0.73 |  |  |  |  |
| Years of professional work(5) | -0.04 | 0.05 | -0.14 | 0.07 | 1 | 0.52 |  |  |  |  |
| Hours per week at WSC(6) | -0.01 | 0.02 | -0.07 | 0.05 | 1 | 0.72 |  |  |  |  |
| Species wolf(7) | 0.14 | 0.10 | -0.08 | 0.37 | 1 | 0.25 |  |  |  |  |
| Sex male(8) | -0.05 | 0.09 | -0.27 | 0.17 | 1 | 0.62 |  |  |  |  |
| (1) not indicated because of having a very limited interpretation | | | | | | | |  |  |  |
| (2) z-transformed to mean of zero and a standard deviation (sd) of one; mean and sd of the original variable were -0.085 and 1.022 | | | | | | | | | | |
| (3) log and then z-transformed to mean of zero and a standard deviation (sd) of one; mean and sd of the original log-transformed variable were 0.985 and 0.943 | | | | | | | | |  |  |
| (4) dummy coded with no being the reference category | | | | | | | |  |  |  |
| (5) log and then z-transformed to mean of zero and a standard deviation (sd) of one; mean and sd of the original log-transformed variable were 1.355 and 0.983, respectively | | | | | | | | | |  |
| (6) z-transformed to mean of zero and a standard deviation (sd) of one; mean and sd of the original variable were 35.915 and 4.559, respectively | | | | | | | |  |  |  |
| (7) dummy coded with dog being the reference category | | | | | | | | | |  |
| (8) dummy coded with F being the reference category | | | | | | | | | |  |

Table S8. Results of the Linear Mixed Model with the behavioral factor II as a response

| Term | Estimate | SE | Lower Cl | Upper Cl | | df | P |  |  |  |  |  |  |  |  |  |
| --- | --- | --- | --- | --- | --- | --- | --- | --- | --- | --- | --- | --- | --- | --- | --- | --- |
| Intercept | 1.45 | 0.10 | 1.23 | 1.65 | |  | (1) |  |  |  |  |  |  |  |  |  |
| Relationship factor(2) | -0.02 | 0.03 | -0.08 | 0.04 | | 1.00 | 0.49 |  |  |  |  |  |  |  |  |  |
| Years of experience with individual(2) | 0.11 | 0.05 | 0.01 | 0.21 | | 1.00 | **0.041** |  |  |  |  |  |  |  |  |  |
| Hand-raiser (yes) | -0.01 | 0.06 | -0.13 | 0.10 | | 1.00 | 0.86 |  |  |  |  |  |  |  |  |  |
| Years of professional work(5) | -0.06 | 0.05 | -0.16 | 0.06 | | 1.00 | 0.30 |  |  |  |  |  |  |  |  |  |
| Hours per week at WSC(6) | 0.00 | 0.02 | -0.06 | 0.05 | | 1.00 | 0.95 |  |  |  |  |  |  |  |  |  |
| Species wolf(7) | -0.72 | 0.12 | -0.94 | -0.46 | | 1.00 | **<0.001** |  |  |  |  |  |  |  |  |  |
| Sex male(8) | -0.13 | 0.09 | -0.32 | 0.09 | | 1.00 | 0.20 |  |  |  |  |  |  |  |  |  |
| (1) not indicated because of having a very limited interpretation | | | | | | | | | | | | | | | |  |
| (2) z-transformed to mean of zero and a standard deviation (sd) of one; mean and sd of the original variable were -0.085 and 1.022 | | | | |  |  |  |  |  |  |  |  |  |  |  |  |
| (3) log and then z-transformed to mean of zero and a standard deviation (sd) of one; mean and sd of the original log-transformed variable were 0.985 and 0.943 | | | | |  |  |  |  |  |  |  |  |  |  |  |  |
| (4) dummy coded with no being the reference category | | | | |  |  |  |  |  |  |  |  |  |  |  |  |
| (5) log and then z-transformed to mean of zero and a standard deviation (sd) of one; mean and sd of the original log-transformed variable were 1.355 and 0.983, respectively | | | | |  |  |  |  |  |  |  |  |  |  |  |  |
| (6) z-transformed to mean of zero and a standard deviation (sd) of one; mean and sd of the original variable were 35.915 and 4.559, respectively | | | | |  |  |  |  |  |  |  |  |  |  |  |  |
| (7) dummy coded with dog being the reference category | | | | | | | | | | | | | | | | |
| (8) dummy coded with F being the reference category  Table S9 Results of the Linear Mixed Model with the behavioral factor III as a response   \| Term \| Estimate \| SE \| Lower Cl \| Upper Cl \| df \| P \| \| \| \| --- \| --- \| --- \| --- \| --- \| --- \| --- \| --- \| --- \| \| Intercept \| 0.94 \| 0.09 \| 0.74 \| 1.13 \|  \| (1) \| \| \| \| Relationship factor(2) \| 0.23 \| 0.05 \| 0.13 \| 0.33 \| 1.00 \| **<0.001** \| \| \| \| Years of experience with individual(2) \| -0.07 \| 0.07 \| -0.20 \| 0.07 \| 1.00 \| 0.36 \| \| \| \| Handraiser (yes) \| 0.11 \| 0.09 \| -0.07 \| 0.30 \| 1.00 \| 0.25 \| \| \| \| Years of professional work(5) \| 0.02 \| 0.06 \| -0.11 \| 0.14 \| 1.00 \| 0.66 \| \| \| \| Hours per week at WSC(6) \| 0.02 \| 0.04 \| -0.06 \| 0.10 \| 1.00 \| 0.68 \| \| \| \| Species wolf(7) \| -0.11 \| 0.10 \| -0.34 \| 0.11 \| 1.00 \| 0.38 \| \| \| \| Sex male(8) \| 0.21 \| 0.09 \| 0.00 \| 0.41 \| 1.00 \| **0.044** \| \| \| \| (1) not indicated because of having a very limited interpretation \| \| \| \| \| \| \| \|  \| \|  \| \| \|  \| \| \| \|  \| \| \| \|  \| \| \| \| \| (2) log and then z-transformed to mean of zero and a standard deviation (sd) of one; mean and sd of the original log-transformed variable were 0.985 and 0.943 \| \| \| \| \| \| \| \| \| (3) dummy coded with no being the reference category \| \| \| \| \| \| \| \|  \| \|  \| \| \|  \| \| \| \|  \| \| \| \|  \| \| \| \|  \| \| \| (4) log and then z-transformed to mean of zero and a standard deviation (sd) of one; mean and sd of the original log-transformed variable were 1.355 and 0.983, respectively \| \| \| \| \| \| \| \| \| (5) z-transformed to mean of zero and a standard deviation (sd) of one; mean and Sd of the original variable were 35.915 and 4.559, respectively \| \| \| \| \| \| \| \| (6) dummy coded with dog being the reference category \| \| \| \| \| \| \|  \| \| \| \| \|  \| \| \| \| \| \|  \| \| \| \|  \| \| \| \|  \| \|  \| \| (7) dummy coded with F being the reference category \| \| \| \| \| \| \|  \| \| \| \| \|  \| \| \| \| \| \|  \| \| \| \|  \| \| \| \|  \| \|  \| \|  \| \| \| \| \| \| \| \| \| \| \|  \| \| \|  \|  \|  \| \| \|  \|  \| \| \|  \| | | | | | | | | | |  |  |  |  |  |  | |
